# Sensitivity analysis for a dynamic macroeconomic policy game in a monetary union

**DOI:** 10.1007/s10100-024-00907-2

**Published:** 2024-03-04

**Authors:** Dmitri Blueschke, Viktoria Blueschke-Nikolaeva, Reinhard Neck

**Affiliations:** Department of Economics, University of Klagenfurt, Universitätsstrasse 65-67, 9020 Klagenfurt, Austria

**Keywords:** Dynamic games, Feedback Nash equilibrium, Pareto solution, Sensitivity analysis, Monetary union, Public debt, Coalitions

## Abstract

In this paper, we examine the sensitivity of the results of an earlier paper which presented and analyzed a dynamic game model of a monetary union with coalitions between governments (fiscal policy makers) and a common central bank (monetary policy maker). Here we examine alternative values of the parameters of the underlying model to show how the earlier results depend on the numerical parameter values chosen, which were obtained by calibration instead of econometric estimation. We demonstrate that the main results are qualitatively the same as in the original model for plausible smaller and larger values of the parameters. For the few cases where they differ, we interpret the deviations in economic terms and illustrate the policies and their macroeconomic effects resulting from the change to the parameter under consideration for one of these cases.

## Introduction

In an earlier paper in this journal (Blueschke et al. 2023b; see also the earlier work Neck and Behrens 2009), we analyzed dynamic interactions in a monetary union with three fiscal players (the governments of the countries or blocks of countries concerned) and a common central bank in the presence of exogenous shocks. The model was calibrated for the Euro Area; it includes a fiscally more solid core block and a fiscally less solid periphery block consisting of two countries (blocks of countries) with different attitudes towards the goal of sustainable fiscal performance. Several coalition scenarios were modelled, namely a fiscal union, a coalition of periphery countries and a coalition of fiscal-stability oriented countries. In addition, a fully cooperative Pareto solution and a non-cooperative feedback Nash (Markov perfect) equilibrium were calculated. The OPTGAME algorithm (Behrens and Neck 2015; Blueschke et al. 2013) was used to determine the approximately optimal time paths of the policy makers’ instrument variables and the resulting paths of the endogenous variables of the model.

One main result of the paper was a ranking of the different scenarios: As expected, the Pareto solution was the best in terms of minimal costs under the given objective functions. This solution concept assumes that full cooperation between all (fiscal and monetary) policy makers prevails and, hence, that the central bank and the governments have agreed to act together over the entire time horizon. This is a strong requirement, which is not realistic in a monetary union as the central bank usually has at least some degree of independence of the fiscal policy makers (and, even stronger, vice versa for the governments). It may serve as a standard of reference for the more realistic noncooperative solutions; by assumption, it outperforms all scenarios with full or partial non-cooperation. Conversely, the fiscal union scenario (cooperation among governments but non-cooperation with the central bank) turned out to cause higher overall costs than the fully non-cooperative and the coalition scenarios. This puts a question mark over endeavors by some politicians in the European Union to institutionalize a common finance minister for the Eurozone and strict cooperation for governments’ fiscal policies.

As the numerical solutions obtained by Blueschke et al. (2023b) were based on roughly calibrated parameters of the macroeconomic model and the objective functions, the generalizability of our results may be questioned. To counter such objections, in the present paper we conduct a sensitivity analysis for all of the parameters of the model, examining the results under the assumptions of a lower and an upper bound for each parameter. An examination of the resulting values of the objective functions shall reveal whether the inferiority of the fiscal union also holds for other plausible values of the parameters, and, if not, under what conditions a fiscal union will be outperformed by non-cooperative fiscal policies. Of course, the results will be contingent upon the dynamic game framework adopted, the particular macroeconomic model, the functional forms and the specification of the model equations, and the assumed objective functions of the policy makers; hence unequivocal policy prescriptions cannot be derived from our analysis. It can show, however, whether the bad performance of a fiscal union is only a numerical artefact or whether it may occur under alternative specifications of the parameters as well. In Sect. 2, we summarize the dynamic game with the objective functions and the macroeconomic model from our earlier paper. Section 3 presents the alternative values of the parameters and the resulting values of the overall objective function under the scenarios investigated. In Sect. 4, we look at the policies and the resulting time paths of the endogenous variables for some cases where the fiscal union outperforms the other (fully or partially) non-cooperative scenarios and provide some interpretations. Section 5 concludes.

## The dynamic policy game

We consider a dynamic tracking game where each player minimizes an objective function (loss function) $J^i$, which is the sum over time of quadratic deviations of state and control variables from given target values (denoted by $\sim$):

1 $$\begin{aligned} \min _{u_1^i,..., u_T^i} J^i= \min _{u_1^i,..., u_T^i}\sum _{t=1}^{T}L_t^i\left( x_t,u_t^1,..., u_t^N\right) ,\hspace{0.5cm} i=1,..., N, \end{aligned}$$

with

2 $$\begin{aligned} L_t^i\left( x_t,u_t^1,..., u_t^N\right) =\left[ x_t-{\tilde{x}}_t^i\right] '\Omega _t^i\left[ x_t-{\tilde{x}}_t^i\right] + \left[ u_t-{\tilde{u}}_t^i\right] '\Psi _t^i\left[ u_t-{\tilde{u}}_t^i\right] . \end{aligned}$$

The game is played over *T* periods and consists of individual optimization problems for *N* players. The penalty matrices $\Omega _t^i$ and $\Psi _t^i$ contain the weights of the deviations of states and controls from their desired levels in any period t and indicate the importance of each of the relevant variables for the players. The players are constrained by a dynamic system of nonlinear difference equations in state-space form:

3 $$\begin{aligned} x_t = f\left( x_{t-1},x_t,u_t^1,\dots ,u_t^N,z_t\right) ,\quad x_0={\bar{x}}_0, \end{aligned}$$

where $x_t$ is an ($n\times 1$) vector of state variables and $u_t^i$ is an ($m_i\times 1$) vector of individual control variables of player *i* ($i=1,..., N$) having $m_i$ variables at their disposal. $z_t$ is a vector of non-controlled exogenous variables including exogenous shocks, $t=1,...,T$. Solutions of the nonlinear dynamic tracking game problem cannot be obtained analytically but have to be numerically approximated using the OPTGAME algorithm (Behrens and Neck 2015; Blueschke et al. 2013). This algorithm allows us to find approximations to cooperative (Pareto optimal) and non-cooperative Markov perfect (feedback) Nash equilibrium solutions of the game.

For our economic application of the dynamic game approach, we consider a model of a monetary union (called MUMOD2) with a common central bank (abbreviated by CB and indexed by E) executing monetary policy for the entire union and three countries or blocks of countries: a core, a thrifty periphery and a less thrifty periphery, with governments 1 (C1), 2 (C2), and 3 (C3), respectively, which are responsible for fiscal policies. The core has a lower initial public debt than the periphery countries; the core and the thrifty periphery attach a higher weight to (low) public debt than the less thrifty periphery.

The common central bank decides on the prime rate $R_{Et}$, a nominal rate of interest under its direct control. The national governments decide on real fiscal surplus (or, if negative, its fiscal deficit), $g_{it}$ ($i = 1, 2, 3$), measured in relation to real GDP. The players use their control variables as instruments in order to track the desired paths of the state variables, which evolve according to the dynamic system. This system is given by the following model:

4 $$\begin{aligned} y_{it}= & {} \delta _i\left( \dfrac{\pi _{jt}+\pi _{kt}}{2}-\pi _{it}\right) -\gamma _i(r_{it}-\theta )+\rho _{ij}y_{jt}+\rho _{ik}y_{kt}-\beta _i\pi _{it}+\kappa _i y_{i,t-1}-\eta _i g_{it}+ zd_{it}, \end{aligned}$$

5 $$\begin{aligned} r_{it}= & {} I_{it}-\pi _{it}^e, \end{aligned}$$

6 $$\begin{aligned} I_{it}= & {} R_{Et}-\lambda _i g_{it} + \chi _i D_{it}, \end{aligned}$$

7 $$\begin{aligned} \pi _{it}= & {} \pi _{it}^e +\xi _i y_{it} + zs_{it}, \end{aligned}$$

8 $$\begin{aligned} \pi _{it}^e= & {} \varepsilon _i\pi _{i,t-1}+(1-\varepsilon _i)\pi _{i,t-1}^e, \hspace{5.0pt}\varepsilon \in [0,1], \end{aligned}$$

9 $$\begin{aligned} y_{Et}= & {} \sum _{i=1}^3\omega _i y_{it}, \hspace{5.0pt}\sum _{i=1}^3\omega _i=1, \end{aligned}$$

10 $$\begin{aligned} \pi _{Et}= & {} \sum _{i=1}^3\omega _i \pi _{it}, \hspace{5.0pt}\sum _{i=1}^3\omega _i=1, \end{aligned}$$

11 $$\begin{aligned} D_{it}= & {} \left( 1+BI_{i,t-1}-\pi _{i,t-1}^e\right) D_{i,t-1}-g_{it}, \end{aligned}$$

12 $$\begin{aligned} BI_{it}= & {} \frac{1}{6}\sum _{\tau =t-5}^t I_{it}. \end{aligned}$$

The meanings of the model variables are presented in Table 1, the parameters of the model are explained in Table 2, and their values in the original paper are shown in Table 3. For details, see Blueschke et al. (2023b).

**Table 1:** Variables of the three-country (*i* = 1, 2, 3) monetary union

*Control variables*	
$g_{it}$	Real fiscal surplus of country *i*
$R_{Et}$	Prime rate
*Endogenous variables*	
$y_{it}$	Short-term deviations from the LR equilibrium output level in country *i*
$r_{it}$	Real interest rate in country *i*
$I_{it}$	Nominal interest rate in country *i*
$\pi _{it}$	Inflation rate in country *i*
$\pi ^{e}_{it}$	Expected inflation rate in country *i*
$y_{Et}$	Weighted output in the monetary union
$\pi _{Et}$	Weighted inflation rate in the monetary union
$D_{it}$	Real government debt in country *i*
$BI_{it}$	Average interest rate for government bonds in country *i*

**Table 2:** Parameters of the three-country (*i*, *j* = 1, 2, 3) monetary union, *x*: target variable

*T*	Terminal period of the game
$\theta$	Natural real interest rate = discount rate of policy makers
$\omega _i$	Weight (share) of country (bloc) *i* in union’s output
$\delta _i$	Impact on output of country *i* of its relative to the average inflation rate in the rest of the union
$\eta _i$	Impact (multiplier) of government budget surplus on output of country *i*
$\varepsilon _i$	Adaptation of current inflation rate to previous period’s inflation rate in country *i*
$\rho _{ij}$	Impact of output in country *j* on output in country *i*
$\beta _i$	Impact of inflation rate in country *i* on its output (size of Pigou effect)
$\gamma _i$	Impact on output of (deviation of) real interest rate (from natural rate) in country *i*
$\kappa _i$	Impact of last period’s output on output in country *i*
$\lambda _i$	Impact of government surplus on nominal interest rate in country *i*
$xi_i$	Impact of output (aggregate demand) on inflation rate in country *i*
$\chi _i$	Impact of government debt (relative to output) on inflation rate in country *i*
$\alpha _{x i}, \alpha _{x E}$	Weight of target variable *x* in country *i*’s policy maker’s (*E*: central bank’s) objective function

The model is essentially a Keynesian demand-side model, with an income-expenditure equilibrium Eq. (4), an expectations-augmented Phillips curve (7) with adaptive expectations (8), and a government budget constraint (11). The exogenous variables $zd_{it}$ and $zs_{it}$ denote demand and supply shocks, respectively. We assume the players of the game (the central bank and the three governments) to aim at minimizing their quadratic intertemporal objective functions, which are given by

13 $$\begin{aligned} J_i= & {} \frac{1}{2}\sum _{t=1}^{T}\left( \frac{1}{1+\theta }\right) ^t\left\{ \alpha _{\pi i}\left( \pi _{it}-{\tilde{\pi }}_{it}\right) ^2+\alpha _{yi}\left( y_{it}-{\tilde{y}}_{it}\right) ^2+\alpha _{Di}\left( D_{it}-{\tilde{D}}_{it}\right) ^2+\alpha _{gi}g_{it}^2\right\} \end{aligned}$$

14 $$\begin{aligned} J_E= & {} \frac{1}{2}\sum _{t=1}^{T}\left( \frac{1}{1+\theta }\right) ^t\left\{ \alpha _{\pi E}\left( \pi _{Et}-{\tilde{\pi }}_{Et}\right) ^2+\alpha _{yE}\left( y_{Et}-{\tilde{y}}_{Et}\right) ^2+\alpha _{E}\left( R_{Et}-{\tilde{R}}_{Et}\right) ^2\right\} \end{aligned}$$

We assume (see Table 4) that the governments put higher emphasis on (national) output while the central bank gives a higher weight to (union-wide) inflation. Moreover, country 1 and country 2 (oriented towards fiscal stability) attach a higher weight to the public debt target ($\alpha _D$) than country 3 (the less thrifty periphery block). For the desired paths of the objective variables (Table 5), we assume that a balanced growth path (the natural level of real GDP) is targeted by all players, i.e. the short-run output gap $y_i$ ($i = 1,2,3$) should be zero. The target value for the inflation rate is set to $2\%$, which corresponds to the official objective of the ECB. Regarding the public debt target, the governments aim to fulfill the Maastricht criteria of $60\%$ of GDP, taking different initial values into account. The governments prefer a balanced budget and the central bank aims at a prime rate of $3\%$. The target values and the weights given to the policy instruments of each player partly reflect the desire to avoid too excessive a fluctuation in these variables. For the cooperative Pareto scenario, the joint objective function is given by the equally weighted sum of the four objective functions:

15 $$\begin{aligned} J=\mu _1J_1 + \mu _2J_2 + \mu _3J_3 +\mu _EJ_E, \hspace{0.5cm} (\mu _1=\mu _2=\mu _3=\mu _E=0.25). \end{aligned}$$

Using the dynamic system (4)–(12) and the objective functions (13)–(15), we analyze growth, fiscal stability and price stability trade-offs in a monetary union in the presence of exogenous shocks. The initial calibration of the parameters of the game, done in an attempt to follow the history of the recent crises in the Euro Area, is shown in Tables 3, 4, 5, 6, and 7 below for the parameters of the economic model MUMOD2, the objective functions and the exogenous shocks. The calibration of the latter aims at reflecting the global effects of the Great Recession 2008–2010, the Euro Area sovereign debt crisis 2011–2014, the COVID-19 pandemic crisis 2020–2022, and the Russian war in Ukraine 2022–2025 (hoping that this will be over by then).

**Table 3:** Parameter values for an asymmetric monetary union, $i=1,2,3$

						$$\rho _{23},\rho _{32}, \beta _i,$$			
*T*	$\theta$	$\omega _1$	$\omega _2,\omega _3$	$\delta _i,\eta _i,\varepsilon _i$	$\rho _{21},\rho _{31}$	$\gamma _i,\kappa _i,\lambda _i$	$\rho _{12},\rho _{13}$	$\xi _i$	$\chi _i$
30	0.03	0.6	0.2	0.5	0.375	0.25	0.125	0.1	0.0125

**Table 4:** Weights of the variables in the objective functions

$\alpha _{yi}, \alpha _{gi}$	$\alpha _{\pi E}$	$\alpha _{yE},\alpha _{\pi i}$	$\alpha _{D1},\alpha _{D2}$	$\alpha _{D3}$	$\alpha _{RE}$
1	5	0.5	1	0.1	3

**Table 5:** Target values for the asymmetric monetary union

${\tilde{D}}_{1t}$	${\tilde{D}}_{2t},{\tilde{D}}_{3t}$	${\tilde{\pi }}_{it}$	${\tilde{\pi }}_{Et}$	${\tilde{y}}_{it}$	${\tilde{y}}_{Et}$	${\tilde{g}}_{it}$	${\tilde{R}}_{Et}$
60	80$\searrow$60	2	2	0	0	0	3

**Table 6:** Modelling the financial crisis and the sovereign debt crisis

*t*	1	2	3	4	5	6	7	8	9	.
*year*	’08	’09	’10	’11	’12	’13	’14	’15	’16	.
$zd_{1t}$	−1	−6	−1	0	0	0	0	0	0	0
$zd_{2t}$	−1	−6	−1	−3	−4	−3	−1	0	0	0
$zd_{3t}$	−1	−6	−1	−3	−4	−3	−1	0	0	0

**Table 7:** Modelling the Covid-19 crisis and the Ukraine war crisis

*t*	12	13	14	15	16	17	18	19	20	21	.
*year*	’19	’20	’21	’22	’23	’24	’25	’26	’27	’28	.
$zd_{1t}$	0	−5	0	0	0	0	0	0	0	0	0
$zd_{2t}$	0	−5	0	0	0	0	0	0	0	0	0
$zd_{3t}$	0	−5	0	0	0	0	0	0	0	0	0
$zs_{1t}$	0	0	1	9	3	2	1	0	0	0	0
$zs_{2t}$	0	0	1	9	3	2	1	0	0	0	0
$zs_{3t}$	0	0	1	9	3	2	1	0	0	0	0

We model the Great Recession crisis and the Euro Area sovereign debt crisis as a demand shock, while the other two crises contain elements of both demand and supply shocks. The sovereign debt crisis affects the core and the periphery countries differently, while the other shocks have symmetric effects on all economies in the monetary union. In order to analyze the effects of different coalition strategies in a monetary union in the presence of negative exogenous shocks, we constructed five scenarios with different coalitions. We take a coalition to mean a strictly binding agreement between two or more players to always act jointly, i.e. the participants in the coalition give up their own objective function and play as one player (following a cooperative strategy within the coalition) with a weighted joint objective function. These scenarios are summarized in Table 8.

**Table 8:** Coalition strategies when facing negative exogenous shocks

	Scenario	Game strategy
sc1_NF4	Everyone for themselves	Nash FB with 4 players
sc2_2+3	Core versus periphery	Nash FB with 3 players
	Coalition of periphery countries	CB / C1 / (C2+C3)
sc3_1+2	Thrifty versus thriftless	Nash FB with 3 players
	Coalition of countries 1 & 2	CB / (C1+C2) / C3
sc4_FU	Fiscal union	Nash FB with 2 players
	Coalition of 3 countries	CB / (C1+C2+C3)
sc5_P	Fiscal and monetary union	Pareto solution
sim	Non-controlled simulation	Simulation

The results of the different scenarios can be summarized as follows; for details, see Blueschke et al. (2023b):

- The policy instruments are used in a countercyclical way during the demand shocks, but monetary policy and mostly also fiscal policy return faster to their long-run course than was actually the case during the crises. On the contrary, neither monetary nor fiscal policy react to the supply-side shock.
- In terms of the value of the overall objective function (Eq. 15), the cooperative Pareto solution results in the best performance and the fixed-rules simulation in the worst, as must be the case. In the fully cooperative solution, monetary policy accommodates the fiscal policies of the governments, and all countries perform much better in terms of both output and public debt than in any of the other scenarios.
- The less thrifty periphery country runs much higher fiscal deficits than the others, which leads to unsustainable public debt levels in all scenarios other than the cooperative Pareto solution. By contrast, the thrifty periphery and the core are able to stabilize their public debt at sustainable levels even after four consecutive crises in the last 14 years, mostly by creating primary surpluses for their budgets in the crisis-free years.
- The pure fiscal union scenario without accommodating monetary policy gives the worst solution in terms of the total objective function value. In this case, the fiscal players have to run very high budget deficits to deal with the effects of the shocks. This result came as a bit of a surprise, although we know that in general partial cooperation may be counterproductive in a strategic game. The question is how robust this result is with respect to variations in the parameters of the game. This is the main motivation for the following analysis.

## Sensitivity analysis

For the analysis of the sensitivity of the results, especially of the inferiority of the purely fiscal union, we ran all scenarios with different values for the parameters of the model and the objective functions. We always kept all parameters except for one as in the original specification (for all of the countries in the case of the model parameters) to find out the influence of each parameter on the qualitative results. In each case, we took one smaller and one larger parameter value, both of which were sufficiently different from the one in the original analysis and were assumed to be unlikely but not impossible. To be more precise, we took 50 percent and 150 percent of the parameter concerned as alternatives for the base value from Tables 3 and 4. To obtain insights into the key results, it is most appropriate to look at the values of the overall objective function in each case and compare them across the scenarios for each of the parameters we changed. These results are shown in Table 9.

**Table 9:** Values of the overall objective function (15)

Parameter	b.v	New val. (%)	$J_P$	$J_{NF1}$	$J_{NF2}$	$J_{NF3}$	$J_{NF4}$
$\delta _i$	0.5	−50	1366.05	1936.60*	1983.55	1964.79	2079.99^∘^
		+50	1330.09	1849.83*	1890.56	1866.24	1982.83^∘^
$\eta _i$	0.5	−50	1418.39	1831.91	1835.93	1829.68*	1843.99^∘^
		+50	1288.94	1938.92*	2047.18	2034.46	2279.10^∘^
$\rho _{21},\rho _{31}$	0.375	−50	1293.39	1650.32*	1669.21	1655.92	1701.80^∘^
		+50	1416.69	2166.03*	2258.23	2233.64	2507.03^∘^
$\rho _{23},\rho _{32}$	0.25	−50	1288.14	1674.64*	1692.17	1683.30	1736.82^∘^
		+50	1436.86	2199.58*	2318.60	2256.13	2534.23^∘^
$\rho _{12}, \rho _{13}$	0.25	−50	1344.40	1884.39*	1927.95	1905.02	2020.61^∘^
		+50	1462.86	2128.29*	2220.87	2251.58	2610.88^∘^
$\beta _i$	0.25	−50	1380.67	2009.55*	2073.77	2033.03	2189.11^∘^
		+50	1371.10	1842.61*	1867.08	1855.18	1932.71^∘^
$\gamma _i$	0.25	−50	1385.24	1774.59	1787.50	1771.67*	1829.91^∘^
		+50	1342.11	1965.90*	2015.18	1996.30	2122.07^∘^
$\kappa _i$	0.25	−50	1328.25	1699.39*	1718.03	1702.32	1747.38^∘^
		+50	1409.72	2151.42*	2261.19	2232.41	2551.52^∘^
$\lambda _i$	0.25	−50	1336.84	1908.62*	1966.14	1942.87	2089.26^∘^
		+50	1352.52	1864.34*	1896.27	1875.40	1963.97^∘^
$\xi _{i}$	0.1	−50	1933.22	2578.91	2594.25	2577.32*	2629.67^∘^
		+50	1018.37	1347.26*	1373.78	1355.92	1432.41^∘^
$\chi _{i}$	0.0125	−50	1325.37	1808.48*	1852.58	1830.40	1936.87^∘^
		+50	1373.29	1976.00*	2015.42	1993.93	2111.69^∘^
$\alpha _{D1}, \alpha _{D2}, \alpha _{yi}$	1	−50	1035.03	1263.63*	1279.41	1268.27	1311.69^∘^
		+50	1564.06	2474.50*	2549.61	2513.71	2705.02^∘^
$\alpha _{D3}$	0.1	−50	1342.53	1864.31*	1907.09	1884.32	1994.85^∘^
		+50	1346.09	1900.34*	1944.29	1921.45	2040.48^∘^
$\alpha _{yE}$	0.5	−50	1310.58	1927.86*	1976.03	1951.08	2074.28^∘^
		+50	1376.52	1852.48*	1891.44	1870.21	1977.60^∘^

In Table 9, “b.v.” (second column) denotes the base value according to Table 3 and 4, and “new val.” (third column) denotes the value used for the sensitivity analysis. In Table 9, “$J_P$” denotes the objective function value of the Pareto solution, “$J_{NF1}$” denotes the value of the sc1_NF4 (everyone for themselves) scenario, “$J_{NF2}$” denotes that of the sc2_2+3 (core vs periphery) scenario, “$J_{NF3}$” the sc3_1+2 (thrifty vs thriftless) scenario, and “$J_{NF4}$” the sc4_FU (fiscal union) scenario. In addition, for every variation of the parameters, the symbol * highlights the best (in terms of the objective function value) and ^∘^ the worst among the not fully cooperative (Nash) scenarios.

Looking at the results of the sensitivity analysis, we see that the most unexpected result, the inferiority of the fiscal union, is rather robust with respect to the choice of parameters. In all the experiments of Table 9, the fiscal union has the worst result (the highest value of the overall objective function, i.e. the highest costs) among all scenarios. In particular, the outcome for the fiscal union is nearly always worse than the purely non-cooperative scenario. Essentially the same result was found when we changed the size (the $\omega _i$) and weights (the $\mu _i$) of the countries, making the core and/or the thrifty periphery smaller to show what happens if some thrifty members of the monetary union become thriftless and then make up a majority in the union (Blueschke et al. 2023a). Even in these cases, the thrifty countries can stabilize their public debt.

## When may a fiscal union be preferable?

It is instructive to look at cases where the fiscal union is not outperformed by the other scenarios. For this purpose, we conducted several additional experiments with changed parameters. For a small value (0.1) of $\eta _i$ (the parameter determining the size of the fiscal policy multiplier), we found that some not fully cooperative scenarios (including the fiscal union) result in approximately the same outcome in terms of the overall costs. In this case, fiscal policy is rather ineffective, and it does not matter whether the governments cooperate with each other or not.

**Table 10:** Values of the overall objective function (15)

Parameter	b.v	New val.	$J_P$	$J_{NF1}$	$J_{NF2}$	$J_{NF3}$	$J_{NF4}$
$\theta$	0.03	−50%	1395.60	1972.51*	2014.50	1996.12	2121.24^∘^
		+50%	1330.39	1846.68*	1889.80	1862.06	1965.51^∘^
		0	1483.63	2111.27*	2149.57	2135.48	2267.32^∘^
		0.05	1334.05	1845.26*	1887.82	1858.41	1957.27^∘^
		0.1	1602.78	2135.43	2162.06^∘^	2113.42*	2153.54
		0.2	3432.41	4356.83^∘^	4300.45	4188.12	4055.83*
$\varepsilon _i$	0.5	−50%	909.78	1313.55	1313.50	1303.82*	1326.30^∘^
		+50%	1762.35	2371.41*	2442.79	2408.67	2583.73^∘^
		0	625.49	911.36^∘^	896.17	890.52	877.15*
		0.01	629.46	917.91^∘^	902.64	897.01	883.82*
		0.1	698.50	1018.26^∘^	1004.37	998.43	990.68*
		1	2158.01	2834.83*	2919.68	2874.28	3072.30^∘^

When we assume the parameter $\theta$ (determining the time preference of the governments) to be relatively large (0.1), Table 10 (which uses the same acronyms as Table 9) shows that the worst outcome results from scenario 2 (the coalition of the two periphery countries), with the fiscal union being the second worst. With $\theta =0.2$, the fiscal union even turns out to be the best among the not fully cooperative solutions. This can be interpreted to mean that under a higher time preference rate, a fiscal union may outperform at least some or even all scenarios in which governments are less cooperative. As it is not implausible that governments may discount the future strongly, especially when they do not look beyond their next election date, this exception could be relevant for actual policy making. In particular, an investigation of governments following a policy of political business cycles, in line with the model of Kirchgässner (1983) with a negative time preference within an election period and a positive one across election periods, might be an interesting task for further research.

The most interesting case is that of a small value of $\varepsilon _i$, where the fiscal union comes out best (apart from the cooperative Pareto solution) and the fully non-cooperative scenario worst. This is also shown in Table 10. Consider the case of small $\varepsilon _i$, that is, a slow adaptation of expected to actual inflation. In this case, the fiscal union outperforms the other non-cooperative scenarios (but not the full monetary and fiscal union). This is a situation which is most strongly opposed to the hypothesis of rational expectations. It implies a kind of stickiness in the economic model (bounded rationality of the private sector), which may make fiscal and monetary policies much more effective than with inflationary expectations adapting more rapidly or even immediately. Therefore, the governments may react more strongly to the crises than under more quickly changing inflationary expectations.

**Fig. 1:**
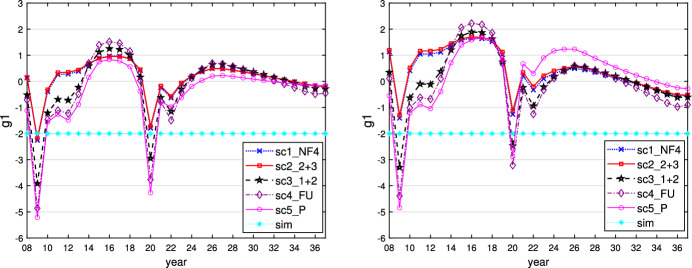
Time path of budget surplus of the core for $\varepsilon _i = 0.1$ (left) and $\varepsilon _i = 0.5$ (right)

**Fig. 2:**
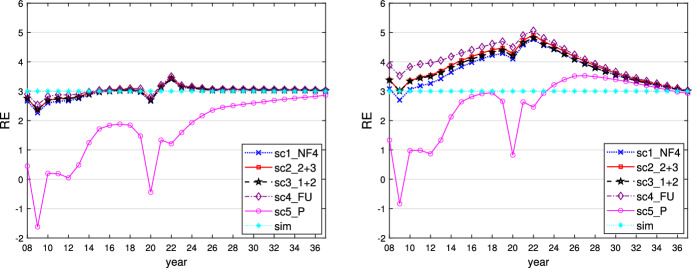
Time path of prime rate for $\varepsilon _i = 0.1$ (left) and $\varepsilon _i = 0.5$ (right)

**Fig. 3:**
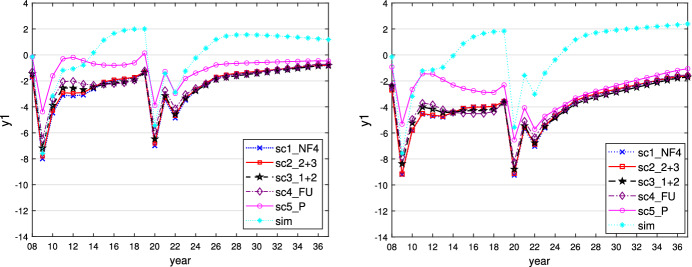
Time path of output of the core for $\varepsilon _i = 0.1$ (left) and $\varepsilon _i = 0.5$ (right)

**Fig. 4:**
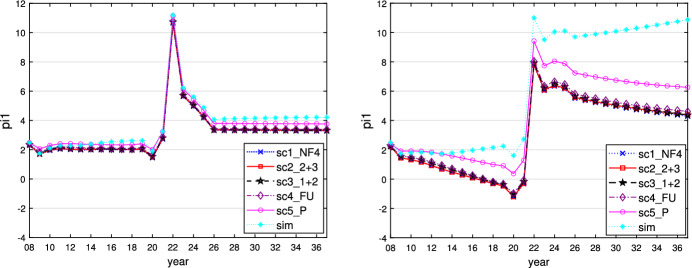
Time path of inflation rate of the core for $\varepsilon _i = 0.1$ (left) and $\varepsilon _i = 0.5$ (right)

**Fig. 5:**
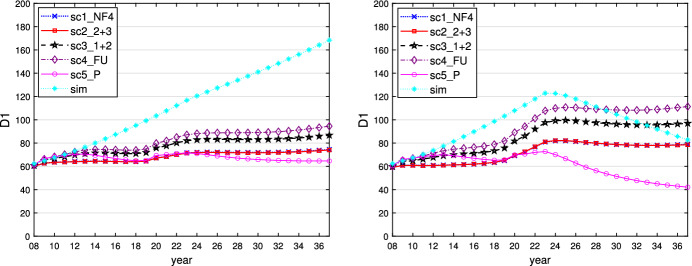
Time path of public debt of the core for $\varepsilon _i = 0.1$ (left) and $\varepsilon _i = 0.5$ (right)

This can be seen when $\varepsilon _i = 0.1$ and is similar for $\varepsilon _i = 0$ or 0.01. We show the time paths of the control variables $g_1$ (similar for $g_2$ and $g_3$) and $R_E$ for $\varepsilon _i = 0.1$ in Figs. 1 and 2. In this case, all governments follow a more active fiscal policy, producing higher budget deficits than in the case where $\varepsilon _i = 0.5$. The restrictive stance of monetary policy is less pronounced here, however, because the inflationary effect of the expansionary fiscal policy is much weaker than in the case of the faster adaptation of inflationary expectations to actual inflation. The task of stabilizing output falls fully on fiscal policies, with weaker negative side effects on price stability, allowing the central bank to act in a less counteracting way vis-à-vis the governments and nevertheless to obtain lower and shorter-lived inflation. As a result of this policy-mix, the fall in output during the crises is lower than in the reference case of $\varepsilon _i = 0.5$, with less increase in public debt because the sticky inflation expectations make real interest rates lower. The time paths of the resulting variables output, inflation rate, and public debt are shown in Figs. 3, 4 and 5.

## Conclusions

We examined the sensitivity of the results for a dynamic game analysis with respect to the parameters of the economic model MUMOD2 and of the objective functions of the players (central bank and governments). The main result is a high robustness of the previous study with respect to the parameters in terms of the ranking of the scenarios investigated. In particular, the overall objective function values show that the full monetary and fiscal union always turns out to be the best and the uncontrolled simulation (corresponding to a regime of fixed rules) the worst institutional arrangement, as expected. The purely fiscal union is dominated by all the other not fully cooperative solutions in the overwhelming majority of the cases investigated, including the solution without any cooperation at all, showing that this (a priori unexpected) result is fairly robust, too. We provided an economic interpretation of a few cases where the fiscal union outperforms the other non-cooperative solutions.

Of course, it would be premature to conclude from this analysis that a fiscal union would be inferior to non-cooperation or partial cooperation in most situations similar to the ones examined here. To obtain more insights into this question, further research will be required. This will include extending the analysis to cover more players (corresponding to the number of countries within the Eurozone), using more sophisticated macroeconomic models, such as DSGE models and/or econometrically estimated models, and a more thorough study of the objective functions of the central bank and the governments. In any case, the possibility of negative effects caused by the governments of a monetary union (including the Eurozone) cooperating in a fiscal union directed at stabilizing the economy without the strong cooperation of the common central bank can serve as a warning against implementing such proposals.
